# Systematic review on the evaluation criteria of orphan medicines in Central and Eastern European countries

**DOI:** 10.1186/s13023-016-0455-6

**Published:** 2016-06-04

**Authors:** Tamás Zelei, Mária J. Molnár, Márta Szegedi, Zoltán Kaló

**Affiliations:** Department of Health Policy and Health Economics, Institute of Economics, Faculty of Social Sciences, Eötvös Loránd University, Pázmány Péter sétány 1/A, 1117 Budapest, Hungary; Institute of Genomic Medicine and Rare Disorders, Semmelweis University, Tömő u. 25-29, 1083 Budapest, Hungary; Syreon Research Institute, Mexikói út 65/A, 1142 Budapest, Hungary

**Keywords:** Rare disease, Orphan drug, Economic evaluation, Technology assessment, Cost effectiveness, Drug approval, Central and Eastern Europe

## Abstract

**Background:**

In case of orphan drugs applicability of the standard health technology assessment (HTA) process is limited due to scarcity of good clinical and health economic evidence. Financing these premium priced drugs is more controversial in the Central and Eastern European (CEE) region where the public funding resources are more restricted, and health economic justification should be an even more important aspect of policy decisions than in higher income European countries.

**Objectives:**

To explore and summarize the recent scientific evidence on value drivers related to the health technology assessment of ODs with a special focus on the perspective of third party payers in CEE countries. The review aims to list all potentially relevant value drivers in the reimbursement process of orphan drugs.

**Methods:**

A systematic literature review was performed; PubMed and Scopus databases were systematically searched for relevant publications until April 2015. Extracted data were summarized along key HTA elements.

**Results:**

From the 2664 identified publications, 87 contained relevant information on the evaluation criteria of orphan drugs, but only 5 had direct information from the CEE region. The presentation of good clinical evidence seems to play a key role especially since this should be the basis of cost-effectiveness analyses, which have more importance in resource-constrained economies. Due to external price referencing of pharmaceuticals, the relative budget impact of orphan drugs is expected to be higher in CEE than in Western European (WE) countries unless accessibility of patients remains more limited in poorer European regions. Equity principles based on disease prevalence and non-availability of alternative treatment options may increase the price premium, however, societies must have some control on prices and a rationale based on multiple criteria in reimbursement decisions.

**Conclusions:**

The evaluation of orphan medicines should include multiple criteria to appropriately measure the clinical added value of orphan drugs. The search found only a small number of studies coming from CEE, therefore European policies on orphan drugs may be based largely on experiences in WE countries. More research should be done in the future in CEE because financing high-priced orphan drugs involves a greater burden for these countries.

**Electronic supplementary material:**

The online version of this article (doi:10.1186/s13023-016-0455-6) contains supplementary material, which is available to authorized users.

## Background

According to the definition of the European Medicines Agency orphan drugs (ODs) are intended for diagnosis, prevention or treatment of rare diseases (RDs), whose conditions affect no more than 5 in 10,000 persons, are life-threatening or chronically debilitating and have no satisfactory method of diagnosis, prevention or treatment [1]. The number of orphan drugs designated by the European Committee of Orphan Medicinal Products (COMP) is expected to grow at an average of 10 % per annum between 2011 and 2020. It seems that the success rate for approvals per orphan designation is also averaged at 10 % [2]. The total number of orphan medicines with marketing authorization is growing year by year [3–5].

The Orphan Drug Act in the United States [6] and the Orphan Drugs Regulation in Europe [7] (also the legislation in Japan and Australia) seem to be a great success. These legislations have generated incentives for the private sector to develop innovative drugs for rare and serious diseases that have had no treatment yet. In some respects, the regulations tried to handle a supply side market failure [8]. Simultaneously, the regulations raised several new dilemmas; one of the most problematic issues being how to apply health technology assessment (HTA) for ODs to support the evidence base of pricing and reimbursement decisions. Owing to the high cost of these medicines and the limitations in clinical evidence, the standard methodology and decision criteria of HTA seem to be difficult to use regarding most of the ODs. Consequently, there is an intensive debate in the scientific literature and also among policymakers and other stakeholders in the everyday practice on how to increase the evidence base of policy decisions without providing disincentives for R&D in rare diseases. There is a need for an adequate, transparent evaluation process to judge the clinical added value of ODs and to provide a consistent decision support tool for policymakers [9].

Financing high-priced orphan drugs is even more controversial in the Central and Eastern European (CEE) region. These middle income countries face even greater challenges in the reimbursement decisions of the ODs, as their financial resources are significantly lower compared to the Western European (WE) countries.

The objective of this systematic literature search was to explore and summarize the recent scientific knowledge about the evaluation and reimbursement of orphan medicines. The research placed special attention on the CEE perspective. There was a special emphasis on the key elements of HTA, including efficacy, effectiveness, cost-effectiveness, budget impact and equity. Additional data was collected about different value drivers in the identified evaluation frameworks. The transferability of the general results to the CEE region will be discussed.

## Methods

A systematic literature search was performed in PubMed and Scopus internet databases to explore recent evidence on value drivers related to the health technology assessment of ODs with a special focus on the perspective of third party payers in CEE countries. The systematic literature review was conducted and reported in compliance with the PRISMA statement [10]. Every relevant record was considered between January 2000 and April 2015. The search terms included rare disease* or *orphan* and reimburse*, evaluation, effective*, assess*, HTA, threshold, decision, policy or *evidence*. Title-Abstract screening was performed with predefined exclusion criteria (see Additional file 1). Special attention was given to papers published in the CEE region. Data extraction occurred in concordance with the main components of HTA, information on efficacy, effectiveness, cost-effectiveness, budget impact and equity were collected in Excel sheets.

The goal of this review was to present a comprehensive overview on any potential value drivers described in the scientific literature, therefore no limitations were applied in terms of the quality of evidence presented by the studies. Editorials, letters, as well as systematic reviews were also included. The type of the articles was registered in every case (see Additional file 2).

Descriptive summaries will be presented in the qualitative synthesis; the nature of gathered information does not allow for a quantitative meta-analysis to be carried out. A further limitation is that the materials presented by authorities, legislative bodies or HTA/technology appraisal committees were not reviewed.

## Results

**Fig. 1:**
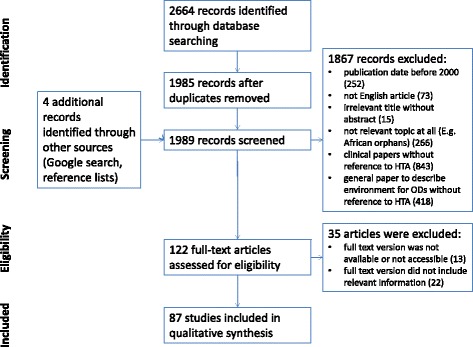
Flow of systematic literature review

All together 2664 items were identified in the two databases from which 1985 records were left after duplicates were removed. Four additional articles were included based on a Google search and after reviewing reference lists in other relevant articles. During the Title/Abstract screening, 1867 records were excluded. The flow of information can be seen on Fig. 1.

After performing a Title/Abstract screening, 122 articles remained for full text screening, which were read and assessed by two reviewers. In the end, 87 full text papers were included in the analysis (see Additional file 2), among which only 5 publications addressed directly the research question from the perspective of CEE countries [11–15].

### Efficacy, effectiveness

**Fig. 2:**
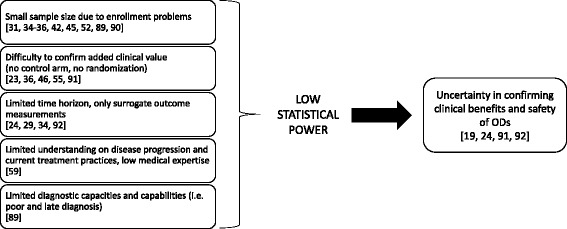
Factors that hinder the efficacy measurement of ODs [19, 23, 24, 29, 31, 34–36, 45, 46, 52, 55, 59, 60, 89–92]

The systematic search found 42 studies that addressed efficacy and effectiveness of ODs from an HTA perspective. In case of orphan medicines, several factors hamper the measurement of the drug’s clinical added value and it seemed difficult to evaluate the drug’s efficacy in the majority of the cases. Figure 2 summarizes the key reasons.

Many of these characteristics had been acknowledged previously and registration authorities realized that along the lines of conventional requirements, market access for ODs would be almost impossible, therefore they softened the registration criteria [16]. As a consequence, the evidence base of ODs in HTA evaluations is often premature, while these drugs receive marketing authorisation earlier in the development phase [17, 18].

Nevertheless, the picture is nuanced: some papers pointed out that in a significant percentage of rare diseases, well-designed clinical trials (e.g., RCTs) would be feasible [19, 20]. It is also possible to modify traditional RCTs designs (sequential, three-stage or adaptive designs) in order to gain more power from a small patient population [21, 22]. Authors emphasized the importance of surrogate endpoints if clinical data are incomplete. These measures can only be accepted and valued in the assessment process if their relation to clinical efficacy is well-described with its uncertainty too [23–25]. At the same time, it seems difficult to validate surrogate markers without a long-term follow up [26].

Despite these facts, the evidence on efficacy should be one of the most important if not the most important value driving component of the technology assessment process [24, 27, 28]. In a recent systematic review, the authors concluded that “ODs [should] have to prove effectiveness like any other drug” [29]. These claims are confirmed by the empirical results of two workshops (experts and patients), which weighted the factor ‘*Evidence of treatment clinical efficacy and patient clinical outcomes*’ with the highest value [30].

A viable option is to declare transparently that more priority is being given to ODs and greater flexibility is being applied for the assessment of forms and/or quantity of available data. A basic remark to this option is that in cases of rare diseases, which are serious or life-threatening, patients are willing to accept a higher level of risk if no other treatments are available [31, 32]. In such cases policymakers may also be willing to accept lower statistical significance of clinical benefits [33].

In case of non-curative OD treatments, the stable or progression-delayed patient status can be a therapeutic benefit. However, the curative and non-curative orphan treatments should be differentiated in medical, ethical and economical aspects [25].

Continuous research after positive reimbursement decisions can be a commitment for the manufacturer (with conditional reimbursement, through observational studies, registries) to generate new evidence with the long-term follow up of patients [34, 35]. Several authors pointed out the usefulness of ongoing evaluations through national and Europe-wide registries of data outcome [24, 36–41].

In summary, the misleading presumptions should be eliminated, the ODs are not a homogenous group, and the feasibility of providing high-quality evidence should be addressed on a case by case basis [42, 43]. Lower standards can only be accepted if substantial efforts failed to gather enough patients in randomized trials [44]. This is the responsibility of registration authorities.

In case of papers referring to the CEE perspective, limited information was available, but the results were similar. Iskrov et al. emphasized in their two papers (2012, 2013) that the absence of clinical data is a common problem for all countries, and especially huge challenge for Bulgaria [11, 12]. The importance of long-term effectiveness measurements was one of the main conclusions of Logviss et al. (2014) also, who analysed the orphan drug situation in Latvia [13].

### Budget impact

The systematic search found 25 studies that addressed the budget impact (BI) of ODs from an HTA perspective. In the majority of countries, BI analysis of ODs is included in the technology assessment process, even though in early years it was not considered a decisive factor due to the low disease prevalence [34]. The relatively small BI facilitated the market access in certain cases [33]. However, due to the escalating drug prices, policymakers started to recognize the growing importance of BI, especially after more and more ODs entered the market. The cumulative expenditure on ODs reached the stimulus threshold of third party payers, and they even expect further increases in the budgets of ODs [2, 12, 29, 32, 33, 41, 45–49]. Based on a projection of Hutchings et al. (2014), BI of ODs in Sweden and France are going to increase slightly until 2020, but it could remain sustainable [50].

Small impact itself cannot be a decisive factor in reimbursement decisions anymore. Usually, opportunity costs of funding rare diseases are not known and therefore cannot be considered comparable with the benefits. As McCabe et al. (2007) stated, the conclusion cannot be made that many small cuts on the budget have no or little impact on overall health or less impact than a single large cut of comparable costs [43]. Instead of the reimbursement of an expensive orphan medication with relatively a small budget impact, it is also a question why not spend the same budget amount on the treatment of a subpopulation in a more frequent disease with a larger cumulative health gain in cases where a medication was rejected earlier because of its high budget impact [28]. The increasing expenditure of ODs challenges the boundaries of the solidarity principle in public healthcare systems because less and less resources are available for treatments of more common diseases [32, 46].

A significant percentage of the overall budget impact is concentrated on only very few drugs. Based on the French data, the first five ‘blockbuster’ ODs account for 50 % of the French OD budget [51]. Overall cost exposure is related to the disease prevalence, the number of indications, the potential for off-label use and the availability of less expensive treatment options. Several authors emphasized that only a detailed budget impact analysis, which includes calculations of the factors mentioned above, would facilitate responsible planning and enable decision makers to develop a sustainable budget for a rare disease portfolio [24, 25, 31, 52, 53]. Ideally, in case of additional indications, the whole budget impact should also be reported [54]. In cases of previously mentioned blockbuster drugs, presumably not all of these elements were carefully considered or the successive reimbursement dossiers of the same orphan drug were not handled in relation to each other [27, 55].

In CEE countries there is stronger restriction of public resources for financing pharmaceuticals. The relatively small market size of CEE countries contributes to their weak negotiating power to influence the price level of ODs, hence prices of these drugs are not among the lowest in Europe as is usually the case with ‘common drugs’. Consequently, the affordability of ODs is more limited in CEE, which reduces the accessibility of patients with rare diseases to ODs [11, 13, 15]. CEE authors pointed out that often there are shortcomings in budget impact analyses, hence the impact should be assessed more accurately [12, 13]. According to Iskrov et al. (2013), in contrast to ‘common drugs’ the legislation in Bulgaria does not clearly define what type of costs should be calculated in budget impact analyses of ODs [12]. In Latvia, the costs related to rare diseases and ODs are currently included in the national healthcare budget, but there are special rules for drugs with a high annual cost, hence the budget for ODs has not been determined explicitly [13].

### Cost-effectiveness

The systematic search found 48 studies that addressed the cost-effectiveness of ODs from an HTA perspective. There is an intensive debate in the literature over how to apply and interpret the cost-effectiveness criteria in the assessment of ODs. The current practice varies across different countries; there are examples where the presentation of a pharmacoeconomic analysis in the reimbursement dossier is not necessary (e.g., Turkey, The Netherlands), in contrast to most other European countries where it is compulsory to perform such an analysis [13, 33, 56–58]. In the past decade, funding for these drugs was rejected more often due to unmet cost-effectiveness criteria [28]. This position was not unified and the issues led to the formulation of a number of questions.

The first problem is the scarcity of good clinical evidence about the effectiveness of ODs, which makes a cost-effectiveness analysis (CEA) difficult. According to some authors, it seems almost impossible to perform a conventional CEA [12, 24, 59]. Even if such an analysis is feasible, only a few ODs get close to meeting the standard cost-effectiveness criterion due to the high incremental costs compared to moderate health gains provided by the drug [45, 46, 48, 60–63]. Cost-ineffective, ultra-orphan drugs have been approved by appraisal committees in several countries, which means that present economic criteria are not sufficient for the evaluation of these drugs, since the true societal value of the provided benefits cannot be measured [42, 43, 64, 65].

Cost-effectiveness thresholds are the symbol of the most highly accepted opportunity cost, in order to determine whether a treatment should be financed publicly or not. Where decisions regarding ODs are systematically made against these norms, these criteria should be revised [66]. If orphan medicines with significantly higher ICERs are routinely reimbursed, then the health gain of rare disease patients are also valued significantly higher. In order to maintain transparency, this preference for severe, rare conditions, which have no other treatment should be explicitly declared [42, 44].

McCabe and colleagues raised an important question: where we insist on applying standard methods to ODs and there is an expectation (as several authors emphasized) that an OD will not receive reimbursement based on pure cost-effectiveness, then a valid question to be asked is why we have incentives to develop them at all. Considering this point, these incentives (previously used resources) are simply sunk costs if the drug is not cost-effective [43, 47]. The most important signal and incentive to the market is the price paid for a drug or the incremental cost-effectiveness ratio used in the evaluation. These values should not be driven by previously provided incentives. If the threshold is transparent, both the evaluation committee and the market know how much money is worth spending on them. The key challenge is the identification of the existence and magnitude of “the orphan premium” [43]. If this can be measured and used transparently, cost-effectiveness threshold could be determined.

One basis of exempting ODs from the standard criteria, if a societal preference could be revealed for prioritizing rarity [61, 63]. A frequently cited study from the NICE Citizen’s Council reported weak evidence about positive social valuation of rarity [67]. Few experimental studies, which investigated this issue, emphasized the choice avoidance and the preference instability of respondents [61, 68–70]. Although experimental research could not confirm any social value, disease rarity alone seems to increase the tolerance of uncertainties by decision makers [44].

In Latvia, there is no specific reimbursement regulation for ODs, but cost-effectiveness analyses are obligatory, therefore the reimbursement decision is value based. Also in Serbia, the assessment committee take the cost-effectiveness of the drug into account [13, 14]. The cost-effectiveness of ODs is not considered in Bulgaria, but the price of ODs should be based on the lowest reference price from a basket of European countries [11].

The literature search found several recommendations in connection with the questions described above. Authors stated that if at least moderate clinical evidence is available, a standard cost-effectiveness analysis can be performed. Certain ODs can be cost-effective along a standard threshold [43, 71]. However, there can be huge uncertainty concerning the estimates that can hinder reimbursement until more evidence becomes available [63]. Policymakers may ignore cost-effectiveness evidence, but in this case, the product must provide the minimal requirements of additional health benefits and at least reliable estimates of budget impact [47, 53]. In opposition to such an approach, several authors suggested an intermediate solution, namely that the standard methodologies of cost-effectiveness analysis for ODs are appropriate, but need to be fine-tuned and updated [29]. Table 1 summarizes the proposed solutions.

**Table 1:** Proposed solution to handle higher ICERs of ODs

Proposed Solution	Description	References
Weighted QALY	*“Weighted QALYs* (according to disease prevalence, severity) *attach a higher value to the health gain of a person with a rare disease. Therefore the ICER will decrease, increasing the likelihood of meeting the* (standard) *threshold.”*	[32, 34–36, 62, 75, 76, 93, 94]
QALY categorization	Prioritization of rare disease groups could be achieved by categorizing QALY’s based on e.g., disease states	[75]
Higher CE-threshold for ODs	Accepting a higher cost-effectiveness threshold for ODs increases the probability that these drugs will be cost-effective	[36, 92–94]
Special rules above the CE-threshold	• Above the cost-effectiveness threshold special support funds or specific political decisions may be needed. • Assess the profitability of ODs on different price levels (cost is warranted and based on a careful consideration of the manufacturer’s cost and returns on investment.)	[95] [47]

No matter how we overrate the health gain of rare disease patients, we contradict the ethical principle for evaluation of people’s lives and health equally [32, 63, 72] (see details in section Equity).

In the CEE region, the question is even more contradictory. The prices are moving in a relatively narrow range across Europe mainly due to external reference pricing, but the cost-effectiveness thresholds in different countries are rather in connection with the gross domestic product (GDP) that differs significantly among the European member states [73]. In several CEE countries, there is an explicit threshold for cost-effectiveness related to the GDP per capita (see Hungary, Poland) or the mandatory base monthly salary (see Slovakia), however rarity and severity of the disease have no impact on the explicit threshold. As there are no specific thresholds for ODs, these medicines are even more rarely cost-effective in CEE [74]. However, a well-defined HTA process with mandatory cost-effectiveness analysis still improves the transparency of policy decisions related to ODs [11].

### Equity

Equity aspects of the reimbursement of ODs are principal, due to the unique position of rare disease patients. All together 37 papers dealt with equity principles and several viewpoints surfaced in the intensive debate surrounding these issues.

The basic moral dilemma is that if we have an extremely high cost - but effective - OD, we will pay for it, despite scarce resources and the possibility of spending the same money on ‘common’ disease patients, where the cumulated health gain would be significantly greater. To put it in question form, should we value the health gain of rare disease patients more highly or in other words should we accept the higher ICERs of ODs?

The utilitarian viewpoint would say ‘No’. According to utilitarians, we have to produce as much good (health) as possible within our fixed limits. Investing substantial amounts of resources for the treatment of rare conditions may be considered unethical from this perspective, because the society’s benefits (and health gain) are not maximized [19, 34, 75]. Additionally, if we pay for orphan medicines with high ICERs, we overrate the health gain of rare disease patients while undervaluing the health gain of common disease patients. This rationale cannot be justified ethically [34]. As the number of reimbursed ODs is growing, even more ‘common’ patients with equal capacity to benefit will be withdrawn from treatments [66]. As long as there is no scientific evidence about societal preference for treating rare and serious disorders, the more frequent serious diseases should be treated where the cumulative health gain is larger [43, 63].

The utilitarian standpoint must be the basis of resource allocation in general, but if we ask, *“Is it fair that people are judged only in terms of how cost-effective their health gain is?”*, and the answer is ‘*No*’ [76], what can be the basis for giving rare disease patients a chance and for paying the premium price of their drugs? Table 2 summarizes literature findings, including the ethical principles cited as a basis for funding orphan medicines, and as it is seen, these are not mutually exclusive. The third column of Table 2 summarizes the main criticisms against each principle.

**Table 2:** Ethical principles that favours price premium of orphan drugs and their criticism

Ethical principle	Description	Critics
“Non-abandonment”	• Society should not abandon individuals who are suffering from a serious and rare condition [19, 96]. • Reimbursement of ODs promotes the appearance of social solidarity where vulnerable groups are supported [35]. • Social justice requires treating everybody with dignity and respect as a human being [97].	• Public healthcare should guarantee the best supportive care for everyone. Restrictions made only for drugs that are far from being cost-effective [8, 44].
“Rule of rescue”	• Society puts greater value on health gains of individuals who are in immediate peril, and there are a small number of cases where no alternative treatments are available [34]. • Identifiable individuals are an essential part of this principle [98]. • Lifesaving ability should be considered in the reimbursement decision, but more specifically, only for therapies of life-threatening diseases, which have no alternative treatments. In this later case, the drugs should be financed irrespectively of their cost [28, 31, 96].	• Immediate, life-threatening peril also characterized several other diseases, for which treatment can be more cost-effective [19, 90]. • Since every person faces imminent death in certain periods of time, this cannot be a differentiating characteristic of rare diseases [8]. • It is not right to select one orphan drug over another as having particular social value, because it is not equal to value lifesaving drugs more than cosmetic drugs [26]. • “Rule of rescue” cannot be feasible at population level in an era of constrained resources [90].
“Rights based approach”	• Social solidarity requires that all members of the society have access to a decent minimum standard of healthcare because it is the right and fair thing to do [92, 97]. • Right of access to high-quality health care is embedded in the legislation of the developed countries [36, 75, 96].	• “Right-based approach” would not necessarily favour the treatment of rare conditions over more prevalent conditions, because these patients also receive the same standard of care [34].
“Equality of opportunity”	• Every member of the society should have the same opportunities to receive treatment and this must be true for rare disease patients as well as other patients with more frequently occurring disorders [60, 96]. • Everybody should have a fair chance to receive not only some treatment, but also the best available treatment [47]. • The equality of opportunity should be the paramount consideration in determining social value [65].	• Effectiveness of ODs is not sufficiently proven in several cases (See section Efficacy, effectiveness)

According to general criticism against ethical principles, there is a propensity to give greater weight to helping identified victims rather than statistical victims. Identified victim bias should be eliminated in case of valuing health gains of rare disease patients [77].

The debate about the position of rare disease patients can be translated into vertical and horizontal equity questions, where those representing ethical considerations generally argue in favor of reasons for vertical equity, while their critics argue in favor of horizontal equity [38].

### Summary of value drivers

The evaluation of orphan medicines should include multiple criteria to appropriately measure the value of these drugs. Paulden et al. (2015) in their systematic review identified several value-bearing factors for ODs [29]. We explored the relevance of these factors from the viewpoint of third party payers in CEE countries, and added other value drivers we identified in the systematic literature review. CEE countries may not necessarily apply all value drivers presented in Table 3, however, this list can be an appropriate basis for the multicriteria evaluation framework for the reimbursement process of ODs. If these criteria are applied in an explicit multicriteria decision analysis (MCDA) tool, selection, scoring and weighting of the criteria may provide a tailored approach for each individual CEE countries. Kolasa et al. (2016) in their MCDA tool developed for the Polish reimbursement process of orphan drugs included ten of these criteria [78].

**Table 3:** Potential value drivers of ODs in CEE countries

Disease-related factors	Treatment-related factors	Economic factors	Societal factors
• Prevalence (rarity) of disease • Severity of disease • Identifiability of the patients of treatment • Loss of QALYs without treatment • Unmet medical need (i.e., availability of treatment alternatives) • Clinical heterogeneity of the disease (i.e., subgroup of patients)	• Evidence of treatment efficacy or effectiveness • Capacity to benefit from the treatment (i.e., magnitude of benefit) • Treatment is curative or delays progression or alleviates symptoms (e.g., palliative care) • Safety profile of treatment • Innovative profile of treatment • Manufacturing complexity	• Cost-effectiveness • Budget impact • Number of indications • Potential for off-label use	• Societal impact of treatment (i.e., indirect costs on families and caregivers) • Equity in access to treatment • Legal considerations (i.e., patent status)

## Discussion

Our systematic search found few studies referring to CEE countries, despite the fact that reimbursement of ODs is becoming increasingly important for public payers in European regions with more economic constraints [79]. Since international evidence on value drivers of ODs is often generated in higher income countries (e.g., Western Europe), the transferability of these research findings to lower income countries has to be considered carefully.

The information on relative effectiveness is considered transferable between higher and lower income countries [80]. Therefore, if the problem is solved by global regulators and policymakers, there is no additional action item from the viewpoint of CEE countries. However, in this region there is even more limited information on the number and distribution of rare disease patients, therefore it is difficult to predict baseline risks and disease progression. In general, the health status of population is lower in CEE compared to WE, so potentially the same relative risk reduction may result in even greater absolute risk reduction and health gain [18]. In addition, adherence and persistence of patients in CEE is even worse than in WE, thus it is difficult to judge how efficacy measured in clinical trials may translate to effectiveness in the real world [81]. Nevertheless, the main objective of third-party payers is to buy health gain. If there is significant uncertainty related to the clinical added value of an OD, it does not make sense to grant reimbursement based on equity to access without considering the efficacy criterion. If initial efficacy results of an OD are very promising in a Phase 2 study, societies may ensure rapid registration, but until the evidence is sufficiently confirmed, the manufacturer has to accept risk-sharing arrangements (e.g., coverage with evidence development) with payers to manage uncertainties related to health outcomes.

Economic principles should be the driver for the allocation of scarce resources [75]. Cost-effectiveness evidence of ODs stems from two different sources, therefore decision should be made on how to manage uncertain estimates of costs and effects [43]. Transferability of cost-effectiveness evidence is highly limited from one country to another, especially if there is a significant difference between the socioeconomic status of the countries. Therefore, the relevance of cost-effectiveness evidence generated in WE is highly limited in CEE, thus local adaptation of international economic models are necessary to draw conclusions on the value of any technologies, including ODs [18].

Presumably, our sense of equity and solidarity form the basis of our willingness to pay a premium price for these drugs. Both equity principles and their counter arguments can be seen as true when examined separately, but if there is an effective and curative drug for a serious, life-threatening disease, everything must be done to finance the price that is placed on the clinical added value of the medicine. There is a tradition to accept equity principles in post-communist CEE countries. The question is how this tradition can be translated to rare diseases, especially in difficult economic periods.

There is an international aspect of equity related to the accessibility of patients to ODs in higher vs. lower income European countries. Within the European Union, there should not be differences between member states in the accessibility of patients with rare diseases to medicines. However, in CEE the public health budgets are particularly limited and vulnerable [11]. Unfortunately, external price referencing may prevent manufacturers of ODs from launching their products at significantly lower prices in lower income European countries [82]. Consequently, the deviation of OD prices is relatively small across Europe, but their affordability is very different across the regions. Systematic research of orphan drug utilization across European countries is rare, however existing information indicates that there are significant differences between WE and CEE countries. O'Mahony et al. (2013) found differences in the availability of treatment and care of hemophilia patients across Europe [83]. Pavlovic et al. (2012) identified inequities in access to ODs between Serbia, Bulgaria and Sweden [14]. Also, Picavet et al. (2012) observed a substantial difference in market uptake of ODs between European countries. They concluded that such variation should have an effect on access to care and should produce a significant inequality of treatment [15]. Logviss et al. (2014) also raised attention to the availability problem of orphan medicines in Latvia. Moreover, those drugs that were available, were often not accessible because of the insufficient reimbursement [13]. Availability and affordability are equally important components of inequities in patient access in EU countries according to EURORDIS [84]. Due to their limited access, the cumulative budget impact of ODs can be similar in CEE and WE, but if CEE countries intend to grant the same access for their patients, the relative impact within the whole budget should be significantly higher. The transferability of budget impact analysis from one country to another is highly limited. If the budget impact of an orphan drug is relatively low in a WE country due to a small patient population, the drug is still not necessarily affordable in CEE.

Equity aspects can justify some price premium, but the magnitude should be acceptable from a societal perspective. And here is a difficult question to raise: is it acceptable that lower income countries have to pay the same high price for ODs on the basis of equity as their wealthier counterparts? Probably not. The value-based price of a new technology is country-specific, usually less in lower income countries and larger in higher income countries, since the willingness to pay for one unit of health gain is different. If European policymakers provide incentives for R&D of ODs to reduce the inequity between patients of common vs. rare diseases, they should develop plans to transfer these benefits to patients in CEE countries. Otherwise, mainly WE patients may enjoy the positive results of EU policies regarding rare diseases. Joint European procurement of ODs might reduce the price of these medicines, and payment according the “ability to pay” by each EU member state can potentially reduce the unequal patient access. However, this solution requires the revision of current EU framework related to external price referencing and parallel trade practices.

## Conclusion

Pricing and reimbursement of ODs should be more transparent and evidence based in the CEE region. Manufacturers should take the budget constraints and the lower ability into account to reimburse these high priced drugs. Policy tools have to be developed to alleviate negative consequences on external pharmaceutical price referencing systems in relation to the accessibility of patients to ODs. Until external price referencing exists and is strengthened even compared to current European practices, manufacturers will not be motivated to implement differential pricing to guarantee faster access of patients in countries with less market potential. The relatively small market potential partially explains why our search found a very small number of studies coming from the CEE region. It is highly unfortunate that policy research is less prevalent in those countries where access to these drugs is more limited. More policy research in CEE is needed in order to take the needs and constraints of these countries into account when developing a European policy framework for ODs.

Current joint policy initiatives by supranational bodies and scientific organizations are promising. The CAVOMP (Clinical Added Value of Orphan Medicinal Products) [85], the IRDiRC (International Rare Diseases Research Consortium), the MoCA (Mechanism of Coordinated Access to Orphan Medicinal Products) Working Group [86], the Rare Diseases Special Interest Group of ISPOR [87] or more broadly the EUnetHTA [88] may provide better evaluation framework for ODs in the near future, which hopefully takes into account the necessities of lower income economies as well.

## Abbreviations

BI, budget impact; CAVOMP, Clinical Added Value of Orphan Medicinal Products; CE, cost-effectiveness; CEA, cost-effectiveness analysis; CEE, Central and Eastern Europe; COMP, European Committee of Orphan Medicinal Products; EU, European Union; EUnetHTA, European network for Health Technology Assessment; EURORDIS, European Organisation for Rare Diseases; GDP, gross domestic product; HTA, health technology assessment; ICER, incremental cost-effectiveness ratios; IRDiRC, International Rare Diseases Research Consortium; ISPOR, International Society For Pharmacoeconomics and Outcomes Research; MoCA, Mechanism of Coordinated Access to Orphan Medicinal Products; OD, orphan drug; QALY, quality adjusted life year; R&D, research and development; RCT, randomized controlled trial; RD, rare disease; WE, Western Europe

## Additional files

Additional file 1: Includes the detailed literature search strategy in PudMed and Scopus databases. Furthermore, the document contains the predefined exclusion criteria for Title-Abstract screening of the records. (DOCX 15 kb) Additional file 2: Contains the extracted dataset which summarizes the general characteristics of the included articles and type of articles. Furthermore, separate columns summarizes the main findings of the articles referring to the specific elements of HTA, as well as other factors to consider in terms of public reimbursement. (XLSX 86 kb)
