# Supplementary material for: Systematic review on the evaluation criteria of orphan medicines in Central and Eastern European countries
Source: Orphanet J Rare Dis. 2016 Jun 4;11:72. doi: 10.1186/s13023-016-0455-6 (PMC4893267; doi:10.1186/s13023-016-0455-6)
Supplement: Additional file 1: — Includes the detailed literature search strategy in PudMed and Scopus databases. Furthermore, the document contains the predefined exclusion criteria for Title-Abstract screening of the records. (DOCX 15 kb) [file 13023_2016_455_MOESM1_ESM.docx]

Literature search strategy:

| Database | Search strategy | Date of search | Hits |
| --- | --- | --- | --- |
| PubMed | ((((((rare disease*[Title]) OR orphan[Title])) AND (((((((((reimburse*[Title/Abstract]) OR evaluation[Title/Abstract]) OR effective*[Title/Abstract]) OR assess*[Title/Abstract]) OR HTA[Title/Abstract]) OR threshold[Title/Abstract]) OR decision[Title/Abstract]) OR policy[Title/Abstract]) OR evidence[Title/Abstract])))) | 01/04/2015 | 759 |
| Scopus | ( ( TITLE ( rare disease* ) OR TITLE ( orphan ) ) ) AND ( ( TITLE-ABS-KEY ( reimburse* ) OR TITLE-ABS-KEY ( evaluation ) OR TITLE-ABS-KEY ( effective* ) OR TITLE-ABS-KEY ( assess* ) OR TITLE-ABS-KEY ( hta ) OR TITLE-ABS-KEY ( threshold ) OR TITLE-ABS-KEY ( decision ) OR TITLE-ABS-KEY ( policy ) OR TITLE-ABS-KEY ( evidence ) ) ) | 01/04/2015 | 1905 |

Predefined exclusion criteria during the Title-Abstract screening:

- publication date before 2000
- not English article
- irrelevant title without abstract
- not relevant topic at all: E.g. African orphans, orphan receptors (whose endogenous ligand has not yet been identified)
- clinical papers without reference to HTA (E.g. describe the molecular background or progression of a rare disease)
- general paper to describe environment for ODs without reference to HTA

The screening was performed with EndNote X6.0.1 reference manager software.
